# A Pooled Analysis of Serum Phosphate Measurements and Potential Hypophosphataemia Events in 45 Interventional Trials with Ferric Carboxymaltose

**DOI:** 10.3390/jcm9113587

**Published:** 2020-11-06

**Authors:** Giuseppe Rosano, Ingolf Schiefke, Udo-Michael Göhring, Vincent Fabien, Stefano Bonassi, Jürgen Stein

**Affiliations:** 1 Department of Medical Sciences, IRCCS San Raffaele Pisana, 00166 Rome, Italy; 2 Department of Gastroenterology, Hepatology, Diabetology and Endocrinology, Klinikum St. Georg, 04129 Leipzig, Germany; i.schiefke@eugastro.de; 3 Clinical Development, Vifor Pharma, 8152 Glattbrugg, Switzerland; udo-michael.goehring@viforpharma.com; 4 Biometrics, Vifor Pharma, 8152 Glattbrugg, Switzerland; vincent.fabien@viforpharma.com; 5 Department of Human Sciences and Quality of Life Promotion, San Raffaele University, 00166 Rome, Italy; stefano.bonassi@sanraffaele.it; 6 Unit of Clinical and Molecular Epidemiology, IRCCS San Raffaele Pisana, 00166 Rome, Italy; 7 Department of Gastroenterology and Clinical Nutrition, DGD Clinics Sachsenhausen, 60594 Frankfurt am Main, Germany; j.stein@em.uni-frankfurt.de

**Keywords:** hypophosphataemia, ferric carboxymaltose, iron, IV iron, phosphate, phosphorus, iron supplements

## Abstract

Ferric carboxymaltose (FCM) has been shown to achieve rapid replenishment of iron stores and correction of anaemia in various populations with iron deficiency. A decrease in serum phosphate (PO_4_^3−^) levels, which in most cases is asymptomatic, has been reported with IV iron preparations. Hypophosphataemia (HP) is a known adverse drug reaction with FCM. This post hoc pooled analysis investigates the frequency, duration, risk factors, and clinical signs of HP as reported in interventional clinical trials with FCM. Pooled data from subjects enrolled across 45 clinical trials in different therapy areas were included. A three-step adjudication process was utilised to identify adverse events of HP. Stratified analyses by therapy group and stepwise logistic regression analysis were used to identify predictors of HP. This pooled analysis confirms that FCM is associated with increased rates of serum PO_4_^3−^ lowering, but mean serum PO_4_^3−^ values were seen to recover at Week 4 and further recover at Week 8. Among all subjects receiving FCM therapy (*n* = 6879), 41.4% (*n* = 2847) reached a PO_4_^3−^ nadir value <2.5 mg/dL at any point on study and 0.7% (*n* = 49) reached a nadir <1 mg/dL. Although gastroenterology and women’s health subjects were identified to be at higher risk, occurrence of severe HP (<1 mg/dL [0.3 mmol/L]) following FCM administration was not observed to be common among subjects in these studies. Furthermore, there was no correlation between laboratory serum PO_4_^3−^ values and the occurrence of reported adverse events related to low PO_4_^3−^ levels.

## 1. Introduction

Hypophosphataemia (HP) is uncommon in the general population and is often an incidental finding, detected in 2–3% of hospitalised patients [1]. The normal range for serum phosphate (PO_4_^3−^) is 2.5–4.5 mg/dL in adults (0.81–1.45 mmol/L). Mild, moderate, and severe HP are defined as a serum PO_4_^3−^ level of 2.0–<2.5 mg/dL (0.65–<0.81 mmol/L), 1–<2.0 mg/dL (0.32–<0.65 mmol/L), and <1 mg/dL (<0.32 mmol/L). However, extracellular fluid PO_4_^3−^ levels represent <1% of the total body phosphorus content and are not a good index for PO_4_^3−^ stores in bones or soft tissue [2,3,4]. Mild HP is usually asymptomatic. Mild or moderate HP of short duration generally does not require treatment. Severe cases with PO_4_^3−^ depletion may cause symptoms such as fatigue [5], myocardial depression [6], rhabdomyolysis [7], seizures [8], and haemolytic anaemia [9] and, over time, can ultimately contribute to bone abnormalities such as osteomalacia or rickets [10,11]. Rarely, the most severe cases of HP can be fatal [12,13,14]; respiratory muscle dysfunction or impaired myocardial metabolism and decreased cardiac contractility have been associated with severe HP [6,13,15,16].

Hypophosphataemia is a known adverse drug reaction with ferric carboxymaltose (FCM) listed in the Ferinject^®^ (FCM) Prescribing Information since registration of the product [17], and it is often transient and, in most cases, asymptomatic. A decrease in serum PO_4_^3−^ has also been reported with several other IV iron preparations [18,19,20]. However, the potential clinical relevance of a decrease in serum PO_4_^3−^ has been challenging to establish due to the generalised symptoms of HP. The mechanisms of FCM-induced HP are still to be fully elucidated, although it is probably attributed to the intracellular metabolism of fibroblast growth factor 23 (FGF23). Initially attributed to regulating PO_4_^3−^ and calcium homeostasis, recent studies have also linked FGF23 to iron homeostasis [21,22,23,24] potentially leading to HP via increased PO_4_^3−^ loss in the urine [21]. Although replenishing iron stores appears to reduce the production of FGF23, IV iron preparations such as FCM may potentially inhibit the proteolytic cleavage of FGF23, thereby increasing the circulating levels of iFGF23 [25], which in a downward cascade, may have an impact on phosphate homeostasis causing phosphaturia. The exact mechanisms mediating iFGF23 increase by FCM is unknown [26].

FCM has been studied extensively in both the clinical trial and real-world settings (>12 million patient-years of post-marketing exposure) [27] and is an effective and generally well-tolerated treatment to rapidly replenish iron stores and correct anaemia in patients with iron deficiency of various aetiologies [28]. FCM is an intravenous iron preparation in which a non-dextran, stable carbohydrate shell facilitates iron release in a controlled manner. As a result, a single high-dose course of FCM (up to 2 × 750 mg of iron in the USA and 1 × 1000 mg of iron in Europe) can be administered over 15 min, allowing rapid iron repletion even in severely iron-deficient patients.

FCM has been shown to achieve rapid replenishment of iron stores and correction of anaemia in various populations with iron deficiency and to be more effective than oral iron therapy or iron sucrose [29,30]. It is generally well tolerated, with a lower risk of severe hypersensitivity reactions compared with some other IV iron formulations [29,31,32,33].

The purpose of this post hoc pooled analysis is to investigate the incidence and extent of HP in FCM-treated patients and to characterise the frequency, duration, risk factors, and potential clinical signs of HP as reported in interventional clinical trials.

## 2. Methods

Analyses were based on the pooled study populations of all clinical trials with FCM that had individual patient data filed in the clinical databases of Vifor Pharma and licensing partners (data lock point: 31 October 2017) [27]. Study duration varied from 1 to 52 weeks. Observational studies and studies without a control group were excluded. For studies with a cross-over design, only data from the first treatment period were analysed. The list of all studies contributing data to the pooled analysis is reported in Supplementary Table S1. Serum PO_4_^3−^ levels were defined according to Common Terminology Criteria for Adverse Events (CTCAE) thresholds (v4.0) and severity definitions (v5.0) as follows: 2.5–<4.5 mg/dL (normal), 2.0–<2.5 mg/dL (mild decrease), 1–<2.0 mg/dL (moderate), and <1 mg/dL (severe) [34,35].

All individual subjects from the selected studies were pooled and analysed. In total, 15,080 subjects (FCM, *n* = 8245 [54.7%], controls, *n* = 6835 [45.3%]) from 45 clinical trials were included in the safety analysis (safety population). Control groups included subjects who received the following treatments: other IV iron (*n* = 1998), oral iron (*n* = 1621), placebo (*n* = 616), and standard medical care (*n* = 2600). All analyses evaluating the modification of serum PO_4_^3−^ levels throughout study duration included the 6879 subjects with post-baseline serum PO_4_^3−^ measurements (83.4% of all subjects receiving FCM).

Stratified analyses were performed by nephrology, cardiology, gastroenterology, neurology, women’s health, and other therapeutic areas. Variables considered to be potential confounders or effect modifiers of the possible relationship between FCM and HP included: gender; age (as a continuous variable categorised as <18, 18–65, ≥65 years); baseline BMI (as a continuous variable categorised as normal, overweight, obese); baseline anaemia (yes/no); baseline ferritin (µg/L); and baseline transferrin saturation (TSAT) (%). Effects of FCM treatment were also considered: single vs. multiple dose (*n* [%]); maximum single dose (categorised as ≤15 mg/kg and >15 mg/kg); and cumulative dose (*n* [%]) (categorised as ≤1000 mg, >1000–≤1500, >1500 mg).

Adjudication of HP adverse events (AEs): investigator-reported AEs containing any of the following MedDRA Preferred Terms (version 20.1) were positively adjudicated as HP: blood phosphorus decreased, hypophosphataemia, hypophosphataemic rickets, and hereditary hypophosphataemic rickets. A set of 318 MedDRA Preferred Terms (including, but not limited to, fatigue, muscle weakness, muscle pain, bone pain, osteomalacia, haemolysis, white cell dysfunction, neurological symptoms, cardiac failure, and ventricular tachyarrhythmia) were interrogated and cross-checked against time to onset, possible treatment, and laboratory PO_4_^3−^ values to adjudicate AEs as HP related to FCM. The algorithm for adjudication of an AE of HP is described in Supplementary Figure S1.

Statistical analysis: All statistical analyses were performed using SAS version 9.4 (SAS Institute Inc., Cary, NC, USA). Mean values of baseline PO_4_^3−^ and corresponding 95% confidence intervals (CIs) were used to determine the extent and timings of change in PO_4_^3−^ from baseline values by plotting residual, leverage, and influence measures as diagnostic quantities.

Univariate logistic regression was first performed to select potential risk factors (*p* ≤ 0.20) then the set of covariates predicting the risk of HP was estimated with a multiple logistic regression model, providing odds ratios and the corresponding CIs for the risk of developing HP associated with FCM treatment.

## 3. Results

### 3.1. Effect of FCM on Serum PO_4_^3−^ Levels

At baseline, 90.4% of patients receiving FCM therapy (*n* = 6879) were anaemic, mean serum ferritin was 43.5 µg/L, mean TSAT was 14.3%, and 98.2% of patients had serum PO_4_^3−^ values ≥2.5 mg/dL (Supplementary Table S2). Baseline mean serum PO_4_^3−^ varied across therapy areas; the proportion of patients with mean PO_4_^3−^ < 2.5 mg/dL ranged from 2.0% (gastroenterology) to 0.6% (non-dialysis-dependent chronic kidney disease (NDD-CKD)). Baseline mean ferritin ranged from 20.2 µg/L (women’s health) and 21.2 µg/L (gastroenterology) to 77.6 µg/L (NDD-CKD) and 109.9 µg/L (haemodialysis-dependent chronic kidney disease (HD-CKD)).

**Figure 1:**
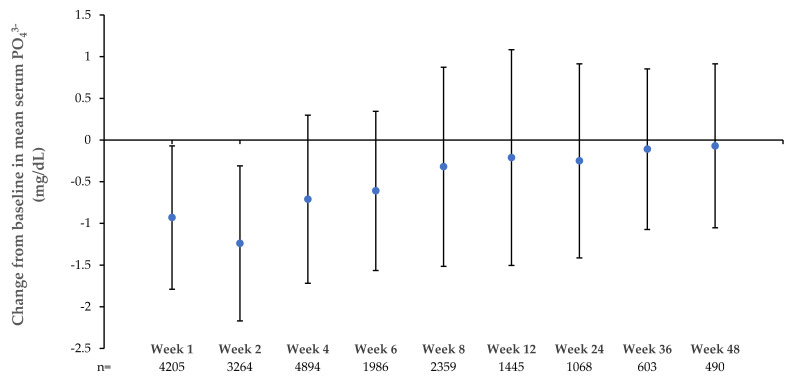
Change from baseline in mean serum PO_4_^3−^ over time in patients treated with ferric carboxymaltose. Patients were included from FCM studies ranging from 1 to 52 weeks in duration. As per individual study protocols, some patients received more than one FCM dose, occurring between Week 8 and Week 48. Subject numbers are indicated for each time point. Bars denote standard deviation of the mean. PO_4_^3−^ reduction was most pronounced at 2 weeks and gradually faded over the following weeks towards baseline values. FCM, ferric carboxymaltose; PO_4_^3−^, phosphate.

Reduction in mean serum PO_4_^3−^ was observed from the first week of treatment with FCM (*n* = 6848), reaching a nadir at Week 2 (Figure 1). This trend was observed to a lesser extent in the other IV iron group but not observed in any of the other control groups (Oral iron, Placebo, Standard Medical Care (Table 1)).

Mean change from baseline in serum PO_4_^3−^ was greatest at 2 weeks; mean baseline PO_4_^3−^ of 3.9 (SD 0.88) mg/dL decreased by 1.24 (SD 0.93) mg/dL, which corresponded to a mean PO_4_^3−^ level of 2.69 (SD 1.08) mg/dL. However, by Week 4 mean serum PO_4_^3−^ levels had recovered to 3.22 mg/dL and recovered further at Week 8 to 3.62 mg/dL.

**Figure 2:**
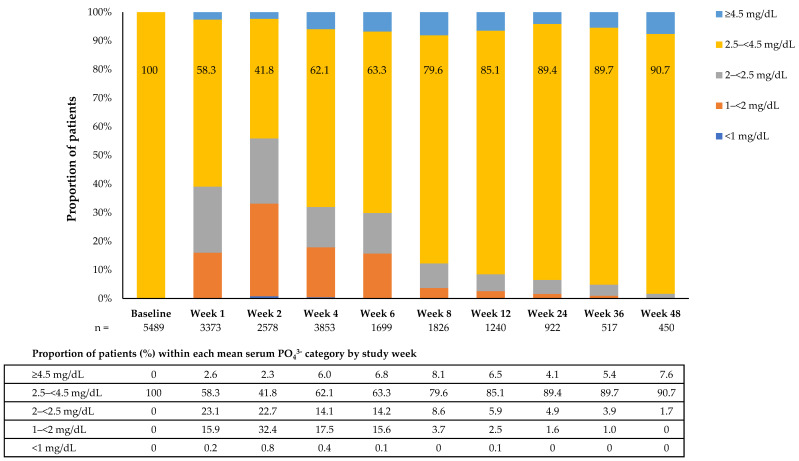
Shift in mean serum PO_4_^3−^ by study week among subjects with normal PO_4_^3−^ values at baseline. Included subjects had baseline PO_4_^3−^ measurements and values 2.5–<4.5 mg/dL (*n* = 5489). Subject numbers are indicated for each time point. Bars and tables depict patients with mean serum PO_4_^3−^ values within each HP range category, as a percentage of all evaluable patients for each study week. HP, hypophosphataemia; PO_4_^3−^, phosphate.

Among all subjects receiving FCM therapy (*n* = 6879), 41.4% (*n* = 2847) reached a PO_4_^3−^ nadir value <2.5 mg/dL at any point during the study and 0.7% (*n* = 49) reached a nadir <1 mg/dL. Of the FCM subjects, 2599 (37.8%) with baseline PO_4_^3−^ 2.5–<4.5 mg/dL developed HP (PO_4_^3−^ <2.5 mg/dL) during the study, with 22.8% of patients reaching values <2 g/dL and 0.7% of patients <1 g/dL at any time point during the studies. Among subjects with normal PO_4_^3−^ values at baseline, 87.7% (1602/1826) of evaluable FCM patients at Week 8 had mean serum PO_4_^3−^ values ≥2.5 mg/dL (Figure 2). This trend was also observed in the total population, with 89.0% of evaluable FCM-treated subjects at Week 8 having mean serum PO_4_^3−^ levels ≥2.5 mg/dL (Table 2). These observations support the hypothesis that FCM treatment induces a temporary decrease in mean serum PO_4_^3−^ levels.

### 3.2. Risk Factors for Hypophosphataemia

The proportion of patients with moderate or severe HP was higher in the gastroenterology and neurology therapy areas (47.1% and 39%, respectively) and considerably less in cardiology, NDD-CKD, and HD-CKD patients (9.9%, 12.3%, and 0%, respectively) than in the total population. Among the 49 patients who reached a PO_4_^3−^ nadir below 1 mg/dL, more than half (55.1%) belonged to gastroenterology studies and 11 (22.4%) to women’s health studies.

**Figure 3:**
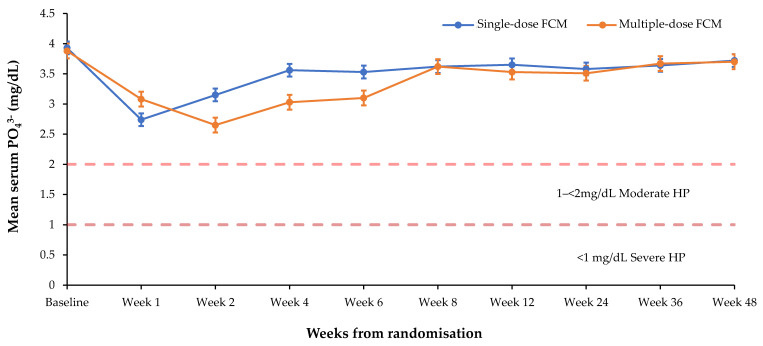
Mean serum PO_4_^3−^ by ferric carboxymaltose dose (single vs. multiple). Single dose *n* = 2808, multiple dose *n* = 4071. Multiple dose is defined as doses given in a minimum of two different administrations. Bars denote standard error of the mean. The drop in serum PO_4_^3−^ was greater and longer lasting after multiple doses compared with a single dose. FCM, ferric carboxymaltose; HP, hypophosphataemia; PO_4_^3−^, phosphate.

**Figure 4:**
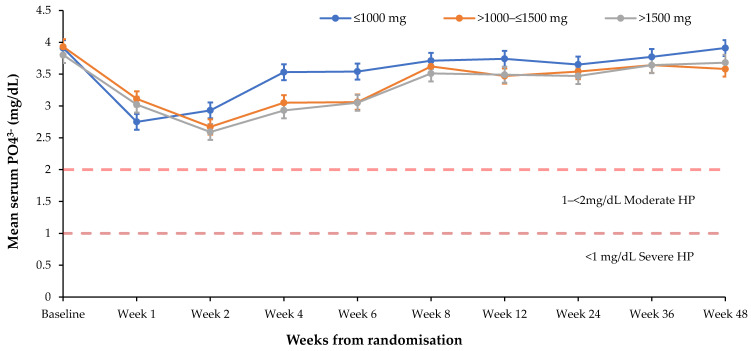
Mean serum PO_4_^3−^ levels over time by cumulative ferric carboxymaltose dose. Cumulative FCM dose ≤1000 mg (*n* = 3131), >1000–≤1500 mg (*n* = 2602), >1500 mg (*n* = 1146). Bars denote standard error of the mean. The drop in serum PO_4_^3−^ was greater in subjects receiving a cumulative FCM dose >1000–≤1500 mg vs. ≤1000 mg. FCM, ferric carboxymaltose; HP, hypophosphataemia; PO_4_^3−^, phosphate.

Serum PO_4_^3−^ levels were lowered to a greater extent and remained low for a longer duration in patients receiving multiple vs. single doses of FCM (Figure 3) (nadir of 2.65 vs. 2.74 mg/dL), in patients receiving frequent (>1 administration during the first 4 weeks of treatment) vs. non-frequent dosing (nadir of 2.61 vs. 3.30 mg/dL), and in patients receiving higher cumulative FCM doses (Figure 4) (nadir of 2.59 mg/dL for >1500 mg vs. 2.67 for 1000–≤1500 mg and 2.75 for ≤1000 mg, respectively. The mean cumulative dose received was 1315 mg (*n* = 6879; min, max: 12.5 mg, 6500 mg).

Risk factors for developing post-baseline moderate HP and post-baseline severe HP within 12 weeks of treatment, identified by logistic regression analyses, included treatment setting, baseline ferritin, and FCM dosing scheme (Table 3). Among the factors tested, the most dominant risk factors for moderate HP only (i.e., subjects who did not progress to severe HP) were therapeutic area (neurology, gastroenterology, and others vs. women’s health), baseline ferritin level, baseline BMI, FCM cumulative dose, FCM single vs. multiple dose, and belonging to Black or African American race. The most dominant risk factors for severe HP were therapeutic area, particularly gastroenterology vs. women’s health (odds ratio (OR): 4.601 [2.093–10.111]), and FCM cumulative dose >1000–≤1500 mg vs. ≤1000 mg (OR: 5.867 [2.514–13.694]). Of note, maximum single dose was not associated with an increased risk of developing severe HP (*p* = 0.522).

### 3.3. Adverse Events Possibly Associated with Hypophosphataemia

#### 3.3.1. Investigator-Reported Adverse Events of HP

Treatment-emergent AEs of HP occurred in 2.2% of subjects receiving FCM (*n* = 179, all non-serious) vs. 0.2% with other IV iron (*n* = 3), 0% with oral iron or placebo, and <0.1% with standard medical care (*n* = 2). Phosphate supplementation was administered to 35 of 179 subjects who had a reported AE of “hypophosphataemia” and “blood phosphorus decreased”. Of note, among 39 subjects with a low laboratory PO_4_^3−^ value at Week 12, one patient required phosphate supplementation.

#### 3.3.2. Clinical Signs Possibly Related to HP

Cross-checking reported AEs against a defined pool of 318 MedDRA terms describing AEs possibly related to HP identified 8.8% (*n* = 726) FCM-treated subjects, 12.5% other IV iron (*n* = 249), 8.6% oral iron (*n* = 140), 14.0% placebo (*n* = 86), and 4.2% standard medical care (*n* = 109) (Table 4). Among all patients with a potential clinical sign of HP, two had a serious treatment-related AE, one (0.01% of the FCM safety population) in the FCM group (congestive heart failure, “other” study group) and one (0.16% of the placebo safety population) in the placebo group (chronic heart failure, “cardiology” study group).

Serious cardiac disorders classified as potentially related to HP occurred in 1.0% FCM (*n* = 81), 1.9% other IV iron (*n* = 37), and 0.6% oral iron subjects (*n* = 10) (Supplementary Table S3). Musculoskeletal and connective tissue disorders occurred in one FCM subject from the NDD-CKD nephrology treatment group (osteoporotic fracture). This occurred in an 80-year-old female and was not positively adjudicated as HP.

Four of 49 FCM subjects (8.2%; 0% serious) who had nadir PO_4_^3−^ below 1 mg/dL had a potential clinical sign of HP (increased C-reactive protein [CRP], fatigue, hypocalcaemia, elevated white blood cell value, swollen finger, tingling in hand) vs. 8.0% (126/1569) with 1–<2.0 mg/dL (0.5% serious), 9.2% (113/1229) with 2.0–<2.5 mg/dL (1.5% serious), and 10.2% (411/4032) with ≥2.5 mg/dL (2.3% serious). These data demonstrate no apparent correlation between laboratory PO_4_^3−^ values and the manifestation of AEs possibly related to HP.

#### 3.3.3. Adjudicated Symptoms among Subjects with Investigator-Reported HP

Adjudicated HP AEs according to treatment type occurred in 0.4% (32) FCM vs. 0.5% (9) other IV iron, 0.4% (6) oral iron, 0% placebo, and <0.1% (1) standard medical care. None of these cases were serious. Within the FCM-treated group, four patients with an adjudicated AE of HP also had serum PO_4_^3−^ levels <1 mg/dL while on-study (two patients within the gastroenterology group and two patients from the women’s health therapy area).

## 4. Discussion

Following FCM administration, HP is a common occurrence [17]; severe HP (<1 mg/dL) and associated severe AEs are not common. The occurrence of severe HP following FCM administration was not observed to be common among subjects in the 45 analysed studies comprising 8245 subjects treated with FCM. Furthermore, there was no correlation between reported laboratory serum PO_4_^3−^ values and the reported occurrence of AEs.

The effect of serum PO_4_^3−^ lowering following administration of FCM has been frequently observed in interventional, observational, and retrospective studies [20,36,37,38,39,40,41,42]. This effect has been seen at 2 weeks following FCM administration, and persistence of laboratory HP at Weeks 5 and 6 has been noted [39,40,41]. Although a greater reduction in serum PO_4_^3−^ levels was observed with FCM vs. comparators in these studies, the shorter study duration of such reports precludes a fuller observation on the duration of this reduction in serum phosphate. The present pooled analysis of clinical studies confirms that FCM is associated with increased rates of serum PO_4_^3−^ lowering, but mean serum PO_4_^3−^ values were seen to recover at Week 4 and further recover at Week 8. Of the 1532 subjects with low serum PO_4_^3−^ levels at Week 2, only 39 such cases remained at Week 12 after the first dose, all of which were limited to mild or moderate levels. Intervention with phosphate supplementation was required for one of these 39 subjects.

Retrospective analysis of individual data from patients enrolled in FCM clinical studies (*N* = 15,080, 8245 of whom were treated with FCM) reconfirms our knowledge that decreases in serum PO_4_^3−^ levels occur more frequently with FCM than with other IV and oral iron therapies. However, these reductions in serum PO_4_^3−^ levels are of a short duration with a nadir at 2 weeks and resolution by about 12 weeks, and a correlation between HP and severe clinical outcomes was not observed. Severe clinical outcomes have been reported in individual case reports, specifically in patients with prolonged exposure to repeated high-dose administrations and pre-existing risk factors for HP (such as underlying disorders causing phosphate malabsorption or vitamin D deficiency) [43,44,45,46,47,48,49,50]. In studies, laboratory findings of HP are generally asymptomatic [28] or adverse drug reactions not observed [41] or clinical outcomes have not been measured [40].

In this analysis, FCM was not associated with an increased rate of adjudicated investigator-reported potential clinical signs/symptoms of HP; no patients with PO_4_^3−^ < 1 mg/dL had adjudicated serious AEs of HP. This observation highlights the apparent lack of correlation between low laboratory PO_4_^3−^ values and the manifestation of AEs considered to be possibly related to HP. Although profound symptomatic HP is rare and often is the manifestation suggestive of a total body phosphate depletion, the investigator-reported AEs in these studied clinical trials did not report serious TEAEs accompanied with chronic HP.

Musculoskeletal and connective tissue disorders occurred in one patient, an 80-year-old female from the NDD-CKD nephrology treatment group. The osteoporotic fracture was reported by the treating physician and was not positively adjudicated as HP; it is to be noted that patients with CKD are prone to fractures due to renal osteodystrophy, which is itself caused by disturbances in metabolic and hormone levels such as parathyroid hormone and vitamin D [51,52]. In our pooled analysis, reported symptomatic HP was rare. For instance, no osteomalacia cases following FCM administration were reported. Treatment with saccharated ferric oxide, iron polymaltose, and FCM have been linked to HP leading to osteomalacia and fractures requiring clinical intervention, including surgery in isolated post-marketing cases concerning patients with anaemia, intestinal ulcer, liver cirrhosis, pneumonia, malabsorption, Crohn’s disease, partial resection of the small intestine, heavy uterine bleeding, and Osler’s disease [53]. Possible signs of symptomatic HP include worsening fatigue with myalgias or bone pain. Post-marketing cases of hypophosphataemic osteomalacia linked to IV iron administration have been observed among patients, mainly with underlying risk factors for HP receiving high cumulative doses or prolonged treatment with FCM [43,46,49] and suggests the cause is multifactorial [43,44,45,46,47,48,49]. Serum PO_4_^3−^ monitoring should be considered in patients who receive multiple administrations at higher doses or long-term treatment, and with existing risk factors for HP.

This analysis study shows that risk factors can be identified at baseline for patients at greater risk of developing HP. Factors indicative of increased risk of moderate HP are found here to include lower BMI and serum ferritin at baseline and administration of FCM dose >500 mg, corroborating existing studies in the area [54,55,56]. The data appear to support an association between higher doses and an increase in the risk of developing severe HP. It is to be noted that this represents only one of the factors contributing to an increased risk and, in practice, represents patients who received a range of doses administered. The mean cumulative dose received was 1315 mg (*n* = 6879; min, max: 12.5 mg, 6500 mg), but administered as varying numbers of doses and with different dosing frequencies. Although a cumulative FCM dose >1000 mg was associated with a trend, there was no maximum single dose associated with an increased risk for developing severe HP. The more prolonged effect on serum PO_4_^3−^ in subjects receiving higher cumulative doses could be due to more and repeated drug administrations. More frequent and multiple FCM administrations, i.e., with shorter time intervals between administrations, have been identified here as being associated with greater reductions in serum PO_4_^3−^ levels. Should HP persist, repeat treatment with FCM should be re-evaluated.

Logistic regression analyses also revealed that the underlying patient therapy area may increase risk of developing HP. The ORs for cardiology and nephrology patients indicate that these groups were at lower risk of developing severe HP following FCM administration, whilst gastroenterology and women’s health subjects were identified to be at the greatest risk. Given that fully functional kidneys have the capacity to modulate excess phosphate in the blood, compromised kidneys (as with CKD patients and heart failure patients with eGFR < 65 mL/min/1.73 m^2^) may have a reduced capacity for renal excretion and therefore are hyperphosphataemic and are at a decreased risk of developing HP following FCM treatment [20]. It has been noted elsewhere that patients with gynaecological causes of iron-deficiency anaemia tend to have higher HP rates, which may explain the higher-than-anticipated incidence of HP following their treatment with FCM [40,42]. Chronic HP has a number of different potential aetiologies, including genetic mutations, altered immunity, tumour induction, and renal disease [57]. Not only the administration of certain drugs, but also comorbidities and medical procedures, including small bowel resection, can contribute to the development of chronic HP. Inflammatory bowel disease patients have a high incidence of metabolic bone disease [58,59], and HP could be an additional risk factor for poor bone health. Given that the trials discussed here were not designed with chronic dosing of FCM, as has been reported with the rare osteomalacia cases in the post-marketing setting, the long-term impact of chronic FCM dosing on bone remodelling markers or markers of renal phosphate wasting was not explored. Inflammatory bowel disease patients may not be at direct risk of developing HP, although confounding factors such as continued malabsorption [60], co-medications [23], vitamin D deficiency [60], and recurrent bleeding episodes predispose this population to lower baseline PO_4_^3−^ values, independent of IV iron therapy. It is therefore recommended to regularly monitor vitamin D and serum phosphate levels as appropriate in patients with underlying disorders caused by vitamin D deficiency or phosphate malabsorption whilst administering iv iron replenishment therapy. In this context, it is helpful to bear in mind that a 75 kg adult has roughly 3–4 g of total iron complement [61]; adequate iron replenishment is, therefore, conventionally achievable with one or two parenteral doses.

This study has several limitations inherent in a retrospective study design: study and patient heterogeneity, variation in observed follow-up, and use of a non-validated three-step algorithm for case adjudication of HP. The observation period reported in this study is longer than has been reported elsewhere but included FCM studies, which varied in duration. Of the 45 included studies, nine ran for ≥24 weeks and two followed subjects for 52 weeks. Seventeen studies were <8 weeks in duration. Cardiology studies tended to have the longest duration, while nephrology studies ranged from 3 to 52 weeks in duration. The consequent fluctuation of subject numbers at each study week should also be considered when interpreting the data presented, although mean values where available for all subjects were utilised, as were single time points and not longitudinal data for each patient.

## 5. Conclusions

This study demonstrates that, although FCM is associated with a reduction in serum PO_4_^3−^ of a short duration with a nadir at 2 weeks and resolution by about 12 weeks, no correlation could be observed between laboratory PO_4_^3−^ values indicative of HP and investigator-reported AEs of HP nor adjudicated cases of HP.

While considering the study limitations, this analysis showed that a temporary and recoverable decrease in PO_4_^3−^ levels is more frequently seen in patients who began therapy with lower ferritin levels and, thereby, required either a higher single or cumulative FCM dose. In this analysis, gastroenterology patients belonged to this profile.

Taken together, these data suggest that although FCM induces the lowering of serum PO_4_^3−^ levels, this is temporary and does not manifest in severe clinical outcomes for the majority of patients across the studied populations. However, in patients identified to be at greater risk of developing HP and potential associated complications, such as patients receiving multiple and/or higher FCM doses or with underlying comorbidities and confounding risk factors, physicians should consider serum PO_4_^3−^ monitoring, while treating these patients with FCM.

## Figures and Tables

**Table 1:** Variation in mean serum PO_4_^3−^ by study intervention with time.

Intervention (*n*)	Mean Serum PO_4_^3−^ at Baseline Mean (SD)	Change from Baseline at Week 2 Mean (95% CI)	Change from Baseline at Week 4 Mean (95% CI)
FCM (6879)	3.90 (0.88)	−1.24 (−1.27–−1.21)	−0.71 (−0.74–−0.69)
Other IV iron (1844)	3.97 (0.81)	−0.28 (−0.31–−0.24)	−0.22 (−0.26–−0.18)
Oral iron (1354)	3.86 (0.67)	0.06 (0.01–0.10)	0.03 (−0.05–0.07)
Placebo (575)	3.61 (0.61)	0.03 (−0.15–0.21)	0.04 (−0.05–0.14)
Standard medical care (2041)	3.93 (0.79)	−0.02 (−0.08–0.05)	−0.05 (−0.08–−0.01)

CI, confidence interval; FCM, ferric carboxymaltose; PO_4_^3−^, phosphate; SD, standard deviation.

**Table 2:** Mean serum PO_4_^3−^ shift from baseline at study Week 8. The numbers of subjects falling within serum PO_4_^3−^ categories at Week 8 are shown by baseline PO_4_^3−^ values. A total of 2371 subjects with baseline measurements had serum PO_4_^3−^ values available at Week 8. “Total” column shows the proportion of subjects in each PO_4_^3−^ value category as a proportion of all available subjects. Grey shaded cells demonstrate subjects with mean serum PO_4_^3−^ values ≥2.5 mg/dL at Week 8 (2112/2371 [89.1%]).

Baseline	<1 mg/dL	1–<2 mg/dL	2–<2.5 mg/dL	2.5–<4.5 mg/dL	≥4.5 mg/dL	Missing Values	Total
Total	0	7	33	1826	493	12	2371/2371 (100%)
<1 mg/dL	0	0	2	0	0	0	2/2371 (0.1%)
1–<2 mg/dL	0	3	6	67	2	2	80/2371 (3.4%)
2–<2.5 mg/dL	0	2	11	157	6	1	177/2371 (7.5%)
2.5–<4.5 mg/dL	0	1	14	1454	249	9	1727/2371 (72.8%)
≥4.5 mg/dL	0	1	0	148	236	0	385/2371 (16.2%)

**Table 3:** Risk factors for developing moderate and for developing severe post-baseline hypophosphataemia as determined by logistic regression analyses. Analysis restricted to subjects with post-baseline serum PO_4_^3−^ measurements (*n* = 6879). Stepwise logistic regression analysis steps are: Step 1: univariate logistic regression performed for each factor. Step 2: multivariate logistic regression for all factors for which *p*-value in Step 1 is ≤0.20. Step > 2: multivariate logistic regression performed excluding the factor that had the highest *p*-value in previous step if it was >0.10. Final step (displayed data): when the *p*-values of all factors included in the model are all ≤0.10. OR and its 95% CI for the selected significant factors is provided.

	Odds Ratio (95% CI)	*p*-Value
**Moderate HP (1–<2.5 mg/dL)**		
Therapeutic area: neurology vs. women’s health	10.274 (0.831–126.95)	<0.0001
Therapeutic area: gastroenterology vs. women’s health	2.728 (2.199–3.385)	<0.0001
Therapeutic area: other vs. women’s health	2.424 (1.925–3.052	<0.0001
Iron parameters: baseline ferritin (10 µg/L increase)	0.922 (0.904–0.940)	<0.0001
Dosing scheme: FCM multiple dose vs. FCM single dose	2.453 (1.615–3.727)	<0.0001
Dosing scheme: FCM cumulative dose >1000–≤1500 mg vs. ≤1000 mg	1.972 (1.311–2.968)	0.0007
Dosing scheme: FCM cumulative dose >1500 mg vs. ≤1000 mg	2.248 (1.483–3.407)	0.0007
Dosing scheme: FCM maximum single dose >500–≤750 mg vs. ≤500 mg	2.279 (1.688–3.077)	<0.0001
Dosing scheme: FCM maximum single dose >750 mg vs. ≤500 mg	1.920 (1.429–2.578)	<0.0001
Intrinsic factors: BMI class (overweight vs. normal)	0.860 (0.724–1.023)	<0.0001
Intrinsic factors: BMI class (obese vs. normal)	0.559 (0.472–0.663)	<0.0001
Intrinsic factors: race (Black or African American vs. White)	2.719 (2.291–3.227)	<0.0001
Intrinsic factors: age <18 years vs. 18–<65 years	0.075 (0.010–0.555)	0.0017
Intrinsic factors: age 65–<75 years vs. 18–<65 years	1.079 (0.845–1.379)	0.0017
Intrinsic factors: age ≥75 years vs. 18–<65 years	1.424 (1.121–1.810)	0.0017
**Severe HP (<1 mg/dL)**		
Therapeutic area: gastroenterology vs. women’s health	4.601 (2.093–10.111)	<0.0001
Therapeutic area: cardiology vs. women’s health	0.259 (0.030–2.228)	<0.0001
Therapeutic area: nephrology vs. women’s health	0.162 (0.032–0.820)	<0.0001
Iron parameters: baseline ferritin (10 µg/L increase)	0.871 (0.742–1.024)	0.0949
Dosing scheme: FCM cumulative dose >1000 mg–≤1500 mg vs. ≤1000 mg	5.867 (2.514–13.694)	0.0002
Dosing scheme: FCM cumulative dose >1500 mg vs. ≤1000 mg	4.093 (1.499–11.180)	0.0002
Intrinsic factors: sex	0.512 (0.249–1.052)	0.0684

CI, confidence interval; FCM, ferric carboxymaltose; HP, hypophosphataemia; OR, odds ratio; SD, standard deviation.

**Table 4:** Rates of adverse events classified as potential clinical signs of hypophosphataemia. AEs possibly related to HP were identified by cross-checking against a set of 318 MedDRA Preferred Terms (including, but not limited to, fatigue, muscle weakness, muscle pain, bone pain, osteomalacia, haemolysis, white cell dysfunction, neurological symptoms, cardiac failure, and ventricular tachyarrhythmia).

Cases with Potential Signs and Symptoms of HP	FCM (*n* = 8245) *n* (%)	Other IV Iron (*n* = 1998) *n* (%)	Oral Iron (*n* = 1621) *n* (%)	Placebo (*n* = 616) *n* (%)	Standard Medical Care (*n* = 2600) *n* (%)
All	726 (8.8)	249 (12.5)	140 (8.6)	86 (14.0)	109 (4.2)
Treatment related	125 (1.5)	36 (1.8)	5 (0.3)	5 (0.8)	19 (0.7)
Severe	132 (1.6)	81 (4.1)	25 (1.5)	19 (3.1)	15 (0.6)
Treatment related	6 (<0.1)	3 (0.2)	0	0	1 (<0.1)
Serious	130 (1.6)	69 (3.5)	32 (2.0)	43 (7.0)	17 (0.7)
Treatment related	1 (<0.1)	0	0	1 (0.2)	0
Leading to withdrawal	23 (0.3)	4 (0.2)	8 (0.5)	10 (1.6)	3 (0.1)
Treatment related	8 (<0.1)	1 (<0.1)	1 (<0.1)	0	2 (<0.1)
Resulting in death	14 (0.2)	6 (0.3)	2 (0.1)	9 (1.5)	2 (<0.1)
Treatment related	0	0	0	0	0

AE, adverse event; FCM, ferric carboxymaltose; HP, hypophosphataemia.

## Supplementary Materials

The following are available online at https://www.mdpi.com/2077-0383/9/11/3587/s1, Table S1: Studies included in the pooled analysis; Table S2: Baseline characteristics of patients receiving FCM therapy; Table S3: Serious treatment-emergent adverse events possibly associated with hypophosphataemia; Figure S1: 3-step hypophosphataemia adjudication algorithm.
