# Supplementary material for: A Pooled Analysis of Serum Phosphate Measurements and Potential Hypophosphataemia Events in 45 Interventional Trials with Ferric Carboxymaltose
Source: J Clin Med. 2020 Nov 6;9(11):3587. doi: 10.3390/jcm9113587 (PMC7694774; doi:10.3390/jcm9113587)
Supplement: Supplementary file 1 [file jcm-09-03587-s001.pdf]

Journal of
Clinical Medicine

Supplementary information

A pooled analysis of serum phosphate measurements and potential hypophosphataemia events in 45 interventional trials with ferric carboxymaltose

Giuseppe Rosano[1]*, Ingolf Schiefke[2], Udo-Michael Göhring[3], Vincent Fabien[4], Stefano Bonassi[5] and Jürgen Stein[6]

1. Clinical Academic Cardiovascular Group, St George's Hospitals NHS Trust, London, UK; giuseppe.rosano@sanraffaele.it
2. Department of Gastroenterology, Hepatology, Diabetology and Endocrinology, Klinikum St. Georg, Leipzig, Germany; i.schiefke@eugastro.de
3. Clinical Development, Vifor Pharma, Zurich, Switzerland; udo-michael.goehring@viforpharma.com
4. Biometrics, Vifor Pharma, Zurich, Switzerland; vincent.fabien@viforpharma.com
5. Department of Human Sciences and Quality of Life Promotion, San Raffaele University, Rome, Italy; Unit of Clinical and Molecular Epidemiology, IRCCS San Raffaele Pisana, Rome, Italy; stefano.bonassi@sanraffaele.it
6. Department of Gastroenterology and Clinical Nutrition, DGD Clinics Sachsenhausen, Frankfurt/Main, Germany; j.stein@em.uni-frankfurt.de

* Correspondence: giuseppe.rosano@sanraffaele.it

Journal of Clinical Medicine

MDPI

Supplementary Table S1 – Studies included in the pooled analysis (n=45). Note that study 1VIT05005 was the extension of study 1VIT04004 and FER-IBD-07-MAIN was the maintenance study of FER-IBD-07-COR.

	Analysis therapeutic area								
	CARDIO	GASTRO	HD-CKD	NDD-CKD	NEURO	Others	Women's Health	Total	Duration
	N	N	N	N	N	N	N	N	Weeks
1VIT03001	.	.	.	.	.	.	174	174	12
1VIT04002	.	.	.	.	.	.	122	122	9
1VIT04003	.	.	.	.	.	.	108	108	9
1VIT04004/5005	.	.	.	199	.	.	.	199	8
1VIT05006	.	113	19	27	.	152	272	583	3
1VIT05009	.	.	.	.	43	.	.	43	24
1VIT06011	.	.	.	.	.	.	142	142	8
1VIT07017	.	.	.	.	.	.	996	996	4
1VIT07018	.	.	50	204	.	.	.	254	4
1VIT08019	.	69	.	40	.	116	118	343	7
1VIT08020	.	31	.	4	.	13	34	82	7
1VIT08021	.	49	.	67	.	129	121	366	6
1VIT08022	.	.	.	.	.	6	18	24	5
1VIT08023	.	.	.	.	.	.	25	25	5
1VIT09030	.	.	.	1276	.	.	.	1276	8
1VIT09031	.	86	.	7	.	131	275	499	20
1VIT13032	.	124	.	70	.	221	91	506	4
1VIT13035	.	102	.	.	.	.	.	102	6
1VIT13036	.	15	.	1	.	16	3	35	5
1VIT14038	.	.	.	.	41	.	.	41	6
BOLINJ-08	.	.	.	.	.	31	.	31	2
FER-AOC-MM	.	.	.	.	.	2	.	2	8

FER-ASAP-2009-01	.	.	.	.	.	.	123	123	12
FER-CARS-01	30	.	.	.	.	.	.	30	26
FER-CARS-02	305	.	.	.	.	.	.	305	26
FER-CARS-03	20	.	.	.	.	.	.	20	26
FER-CARS-04	88	.	.	.	.	.	.	88	24
FER-CARS-05	152	.	.	.	.	.	.	152	52
FER-CKD-01	.	.	.	304	.	.	.	304	52
FER-FID-CHEMO	.	.	.	.	.	8	.	8	8
FER-IBD-07-COR/MAIN	.	294	.	.	.	.	.	294	12
IDNA 2009-01	.	.	.	.	.	.	145	145	10
ThromboVIT	.	16	.	.	.	.	.	16	6
VIRD-VIT-45-IM	.	.	4	.	.	.	.	4	24
VIT-53214	.	.	162	.	.	.	.	162	10
VIT-IV-CL-001	.	.	.	3	.	3	.	6	4
VIT-IV-CL-002	.	.	.	.	.	24	.	24	1
VIT-IV-CL-003	.	46	.	.	.	.	.	46	8
VIT-IV-CL-008	.	137	.	.	.	.	.	137	12
VIT-IV-CL-009	.	.	.	.	.	.	227	227	12
VIT-IV-CL-015	.	.	119	.	.	.	.	119	12
VIT-RLS-2012-013	.	.	.	.	58	.	.	58	12
Z213-01	.	.	.	.	.	24	.	24	1
Total	595	1082	354	2202	142	876	2994	8245	.

CARDIO, cardiology; GASTRO, gastroenterology; HD-CKD, haemodialysis-dependent chronic kidney disease; NEURO, neurology; NDD-CKD, non-haemodialysis-dependent chronic kidney disease.

Journal of Clinical Medicine

MDPI

Supplementary Table S2 – Baseline characteristics of patients receiving FCM therapy. Baseline data available for 6,879 subjects. Laboratory baseline assessments are the last available measurements before the first administration of study drug. FCM frequent dosing is defined as more than one FCM administration during the first 4 weeks.

Parameter	Total patients (N=6,879)
Percentage male [n (%)]	1,437 (20.9%)
Age at consent (years)	n=6,878
<18 years	45 (0.7)
18-<65 years	4,933 (71.7)
65–<75 years	950 (13.8)
≥75 years	950 (13.8)
Missing	1 (<0.1)
BMI (%)	
Normal	1,810 (26.3)
Overweight	1,903 (27.7)
Obese	2,915 (42.4)
Missing	251 (3.6)
Baseline anaemia status	
Anaemic	6,222 (90.4)
Non-anaemic	656 (9.5)
Unknown	1 (<0.1)
Ferritin (µg/L) baseline value	n=6,875
Mean (SD)	43.5 (61.52)
Serum phosphorous (mg/dL) baseline value	n=6,848
Mean (SD)	3.9 (0.88)
<2.5 mg/dL	92 (1.3%)
≥2.5 mg/dL	6,756 (98.2%)
Analysis therapeutic area [n (%)]	
Gastroenterology	734 (10.7%)
Cardiology	578 (8.4%)
NDD-CKD	2,108 (30.6%)

HD-CKD	70 (1.0%)
Neurology	141 (2.0%)
Women's health	2,631 (38.2%)
Other*	617 (9.0%)
TSAT (%) baseline value	n=6,738
Mean (SD)	14.3 (10.36)
FCM dose regimen [n (%)]	
FCM single dose	2,808 (40.8%)
FCM multiple dose	4,071 (59.2%)
FCM maximum single dose (mg) [n (%)]	
≤500 mg	987 (14.3)
>500–≤750 mg	2,837 (41.2)
>750 mg	3,055 (44.4)
FCM frequent vs non-frequent dose [n (%)]	
FCM frequent dosing	3,535 (51.4%)
FCM non-frequent dosing	3,344 (48.6%)
Number of FCM administrations	n=6,879
Mean (SD)	2.2 (2.02)
FCM cumulative dose group (mg) [n (%)]	
≤1,000 mg	3,131 (45.5%)
>1,000–≤1,500 mg	2,602 (37.8%)
>1,500 mg	1,146 (16.7%)

*Others = IDA, oncology, peritoneal dialysis chronic kidney disease, surgery, other bleeding disorder, and unknown or multifactorial reasons for ID/IDA. FCM, ferric carboxymaltose; ID, iron deficiency; IDA iron deficiency anaemia.

Supplementary Table S3 – Serious treatment-emergent adverse events possibly associated with hypophosphataemia. Highlighted rows indicate AE terms which may be more commonly associated with HP. *Blood creatinine increased. Adverse event coding is harmonized to MedDRA Version 20.1.

	FCM (N=8,245)		Other IV iron (N=1,998)		Oral iron (N=1,621)		Placebo (N=616)		Standard medical care (N=2,600)	
System organ class MedDRA Preferred Term	n (%)	Incidence rate (subject) (per 100 PY)	n (%)	Incidence rate (subject) (per 100 PY)	n (%)	Incidence rate (subject) (per 100 PY)	n (%)	Incidence rate (subject) (per 100 PY)	n (%)	Incidence rate (subject) (per 100 PY)
Any adverse event	130 (1.6)	6.48	69 (3.5)	11.83	32 (2.0)	6.07	43 (7.0)	16.08	17 (0.7)	6.28
Cardiac disorders	81 (1.0)	4.04	37 (1.9)	6.34	10 (0.6)	1.90	37 (6.0)	13.84	12 (0.5)	4.44
General disorders and administration site conditions	5 (<0.1)	0.25	1 (<0.1)	0.17	1 (<0.1)	0.19	0	-	0	-
Infections and infestations	0	-	0	-	1 (<0.1)	0.19	0	-	0	-
Investigations*	1 (<0.1)	0.05	0	-	0	-	0	-	0	-
Metabolism and nutrition disorders	1 (<0.1)	0.05	0	-	0	-	0	-	0	-
Musculoskeletal and connective tissue disorders	1 (<0.1)	0.05	1 (<0.1)	0.17	0	-	0	-	0	-
Nervous system disorders	3 (<0.1)	0.15	1 (<0.1)	0.17	2 (0.1)	0.38	0	-	1 (<0.1)	0.37
Psychiatric disorders	2 (<0.1)	0.10	0	-	0	-	0	-	1 (<0.1)	0.37
Renal and urinary disorders	40 (0.5)	1.99	32 (1.6)	5.49	20 (1.2)	3.80	3 (0.5)	1.12	4 (0.2)	1.48
Respiratory, thoracic and mediastinal disorders	4 (<0.1)	0.20	3 (0.2)	0.51	4 (0.2)	0.76	4 (0.6)	1.50	0	-
Surgical and medical procedures	0	-	0	-	0	-	1 (0.2)	0.37	0	-

AE, adverse event; FCM, ferric carboxymaltose; HP, hypophosphataemia; PY, patient-years.

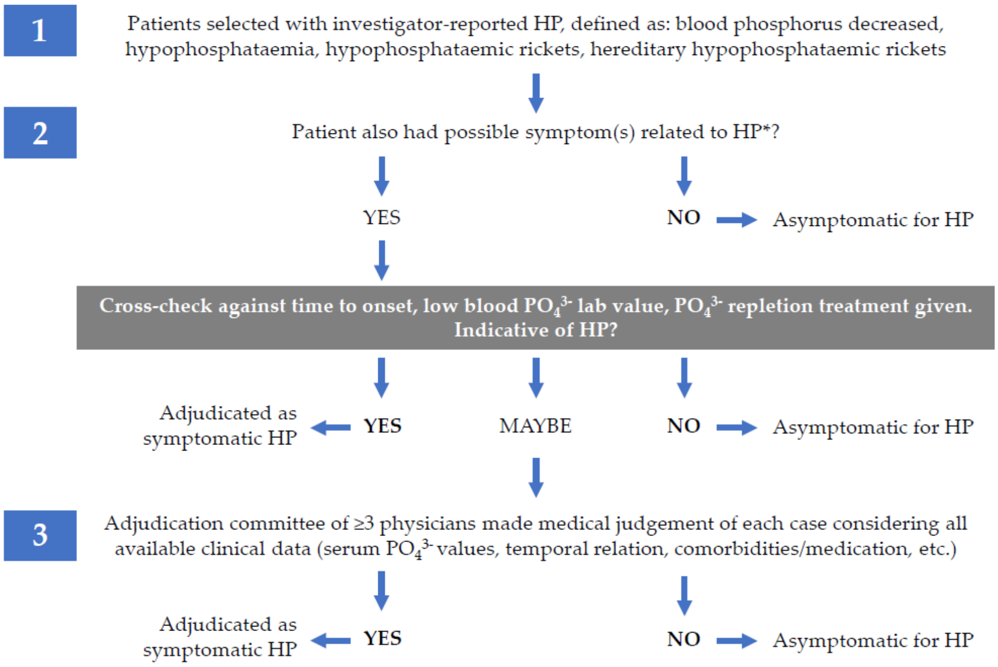

Supplementary Figure 1 – 3-step hypophosphataemia adjudication algorithm. 1) Investigator-reported AEs containing any of the following Preferred Terms were positively adjudicated as HP: blood phosphorus decreased, hypophosphataemia, hypophosphataemic rickets, and hereditary hypophosphataemic rickets. 2) *A set of 318 MedDRA Preferred Terms (including, but not limited to, fatigue, muscle weakness, muscle pain, bone pain, osteomalacia, haemolysis, white cell dysfunction, neurological symptoms, cardiac failure, and ventricular tachyarrhythmia) were interrogated and cross-checked against time to onset, possible treatment and laboratory PO_4^{3-} values to adjudicate AEs as HP related to FCM. Subjects with a positive match were adjudicated as symptomatic HP. 3) Uncertain cases were carried forward to the next stage of adjudication where ≥3 physicians assessed all available clinical data to determine the presence of symptomatic HP in these patients. AE, adverse event; FCM, ferric carboxymaltose; HP, hypophosphataemia; PO_4^{3-}, phosphate.
